# Intervening to enhance collaboration in translational research: A relational coordination approach

**DOI:** 10.1017/cts.2017.10

**Published:** 2017-08-14

**Authors:** Jennifer Perloff, Alice Rushforth, Lisa C. Welch, Denise Daudelin, Anthony L. Suchman, Jody Hoffer Gittell, Hannah Santos, Joanne Beswick, Saleema Moore, Harry P. Selker

**Affiliations:** 1 Brandeis University, Waltham, MA, USA; 2 Tufts Medical Center, Tufts Clinical and Translational Science Institute, Boston, MA, USA; 3 Relationship Centered Health Care, Rochester, NY, USA

**Keywords:** Relational coordination, collaboration, culture, formative evaluation

## Abstract

**Introduction:**

A core challenge of a multidisciplinary and multi-organizational translational research enterprise such as a Clinical and Translational Research Award (CTSA) is coordinating and integrating the work of individuals, workgroups, and organizations accustomed to working independently and autonomously. Tufts Clinical and Translational Science Institute (CTSI) undertook and studied a multifacted intervention to address this challenge and to create a culture of systems thinking, process awareness, responsive to others' needs, and shared decision-making.

**Intervention:**

The intervention, based on relational coordination, included 1) relational interventions, in three staff retreats and a diagnostic survey to provide feedback on the current quality of relational coordination, and 2) structural interventions, in the launching of five new cross-functional teams with regular meeting structures.

**Methods:**

A mixed-methods evaluation yielded quantitative data via two types of team surveys and qualitative data via interviews and meeting observations.

**Results:**

The findings suggest that interventions to improve relational coordination are feasible for CTSAs, including good fidelity to the model and staff/physician engagement. Survey and interview data suggest model improvements in coordination and alignment. Further research about their optimal design is warranted.

## Introduction

Meeting the objectives of clinical and translational research requires the input of many stakeholders including researchers, clinicians, pharmacists, statisticians, information technology staff, educators, institutional review board members, administrators, and others. The National Institutes of Health (NIH) Clinical and Translational Science Award (CTSA) program was founded to break down traditional silos between these groups to foster more collaboration, to produce better science, and to accelerate implementation of biomedical discoveries for the benefit of the public. However, tradition and culture have posed ongoing barriers to collaboration among these stakeholders [1–5]. Lack of communication, differences in education and training, and siloed goals have been identified as sources of these cultural differences [6].

To address the gap between the need for broad collaboration and its realization thus far, Tufts Clinical and Translational Science Institute (CTSI), beginning in 2014, aimed to use the theory and tools of relational coordination (RC) to diagnose and improve collaboration, innovation, and productivity. This paper describes the intervention and its early results.

## Intervention Design

The intervention was grounded in the theory of RC, which posits 7 dimensions through which highly interdependent tasks are most effectively coordinated: communication that is sufficiently frequent, timely, accurate, and focused on problem-solving rather than blaming, and is supported by relationships characterized by *shared goals*, *shared knowledge*, and *mutual respect* [7]. Extensive research suggests that RC strengthens quality, safety, efficiency, customer satisfaction, customer engagement and worker well-being, while promoting learning, innovation, and productivity [8–15]. Moreover, the need for RC is particularly acute under 3 conditions: (1) interdependence, (2) uncertainty, and (3) time constraints [11]. Translational research faces all 3 of these conditions.

**Fig. 1:**
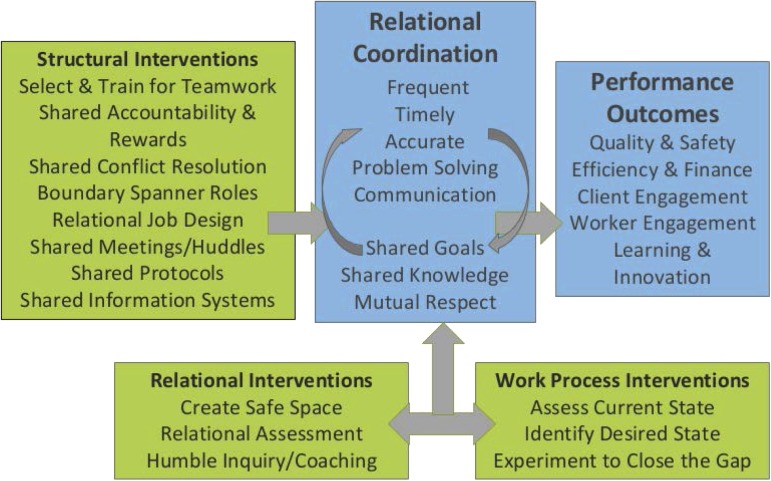
The relational model of organizational change.

Although interventions to improve performance through enhanced RC are at an early stage of development, they typically include the following 3 components shown in Fig. 1 [16].

- Relational interventions that improve the quality of communication, alignment, and shared understanding of the work process from a systems perspective;
- Work process interventions that change how the work itself gets done by collectively assessing the current state, identifying the desired state, and experimenting to close the gap; and
- Structural interventions such as redesigned meetings, boundary spanner roles, staff selection and training, reward and incentive systems, and information systems to ensure that organizational structures intentionally promote RC.

This model of change guided the interventional work carried out by Tufts CTSI. Two target work processes – Education and Research Services – were selected by CTSI leaders for the intervention due to their importance and coordination challenges. Four key components of the intervention are described below.

### Introduction to RC and Relational Mapping

We initiated the intervention with a 1-hour presentation for the Tufts biomedical research community on the principles of RC and demonstrating its association with organizational performance outcomes. Immediately afterwards, we conducted a 2-hour relational mapping workshop for CTSI faculty and staff. We divided participants into groups of 6 and invited each group to draw a diagram identifying the workgroups most involved in the target work processes, both within and beyond Tufts CTSI, and the quality of the RC between them. Their maps showed weak RC ties in some important intergroup relationships, suggesting improvement opportunities. As a relational intervention, relational mapping helped participants begin to view their work from a systems perspective, and it helped to identify which workgroups to include in the RC survey.

### RC Survey

Building on the relational mapping and the early hypotheses generated by participants regarding strengths and opportunities, a baseline survey was administered to a large sample of faculty and staff across all relevant workgroups for a more accurate diagnostic of RC. The survey assessed the quality of communicating and relating between and within workgroups as experienced by all participants and provided data to share with CTSI staff and leadership to develop an intervention. Two additional RC surveys were administered to faculty and staff approximately 1 and 2 years after the baseline survey to monitor RC and allow for mid-course modification of the intervention. Survey results were shared with workgroup members in the form of short written reports or presentations.

### Workshops for Reflective Debriefing

At two 3-hour Reflective Workshops, 1 each for the Education and Research Services work processes, baseline RC survey results were shared with participants in order to identify the most promising opportunities for improvement. Participants in both workshops identified shared knowledge and timely communication as the most important RC dimensions in need of improvement. Participants then engaged in 2 activities to enhance RC: a goal-mapping activity for assessing and improving alignment (shared goals), and “conversations of interdependence,” a set of questions that promote lateral feedback about each other’s work (shared knowledge) [16]. Participants then developed action plans for moving forward. These workshops were in effect relational interventions that led to identification of structural interventions as described below.

### Coalition Meetings

**Fig. 2:**
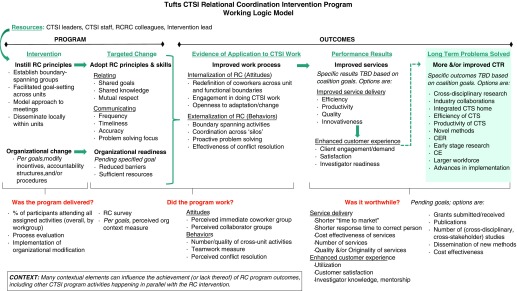
Tufts Clinical and Translational Science Institute (CTSI) relational coordination (RC) intervention logic model. CE, community engagement; CER, comparative effectiveness research; CTR, clinical and translational research; RCRC, relational coordination research collaborative; TBD, to be determined.

Following the Reflective Workshops, Tufts CTSI leaders undertook a major structural intervention, creating 3 cross-functional coalitions and 2 cross-functional taskforce teams with the goal of enabling workgroups to get their own work done faster and to extend their capacity to do new, innovative, and interdependent work with others in Tufts CTSI. These new teams included representatives from different CTSI workgroups within the Education and Research Services work processes. For the most part, the members of these cross-functional teams had not previously met on a regular basis. A logic model was developed to define target changes and outcomes (Fig. 2).

At the kick-off meeting each team collectively defined the purpose of the coalition or taskforce, laying the groundwork for identifying projects of joint interest to cross-functional team members. The Education Coalition was responsible for improving coordination, resource sharing, building new programs, and fostering mutual support among 3 distinct research education programs within Tufts CTSI. The Research Services and Clinical Trials Coalitions were both derived from workgroups within the Research Services work process. These coalitions were charged with improving the comprehensiveness, integration, and utilization of research support services offered to investigators by Tufts CTSI. Two other cross-functional groups, the Navigators Taskforce and the Community Taskforce, were also formed to enhance administrative capabilities and community outreach, respectively. Although the Education, Research Services, and Clinical Trials Coalitions were the target of evaluation described here, the 2 taskforce groups adopted the intervention practices and helped to promulgate RC principles throughout the organization.

The coalitions were intended to be vehicles for a culture change within Tufts CTSI that would include more open perspective sharing, responsiveness to each other’s needs, shared decision-making, and mindfulness of and accountability for group process. Two Tufts CTSI leaders were trained and coached by the external consultant for 5–6 hours in the use of specific meeting management tools to promote the intended culture change, such as ground rules, check-ins, shared agenda setting, nominative group process, reflective time-outs, and appreciative debriefings.

**Fig. 3:**
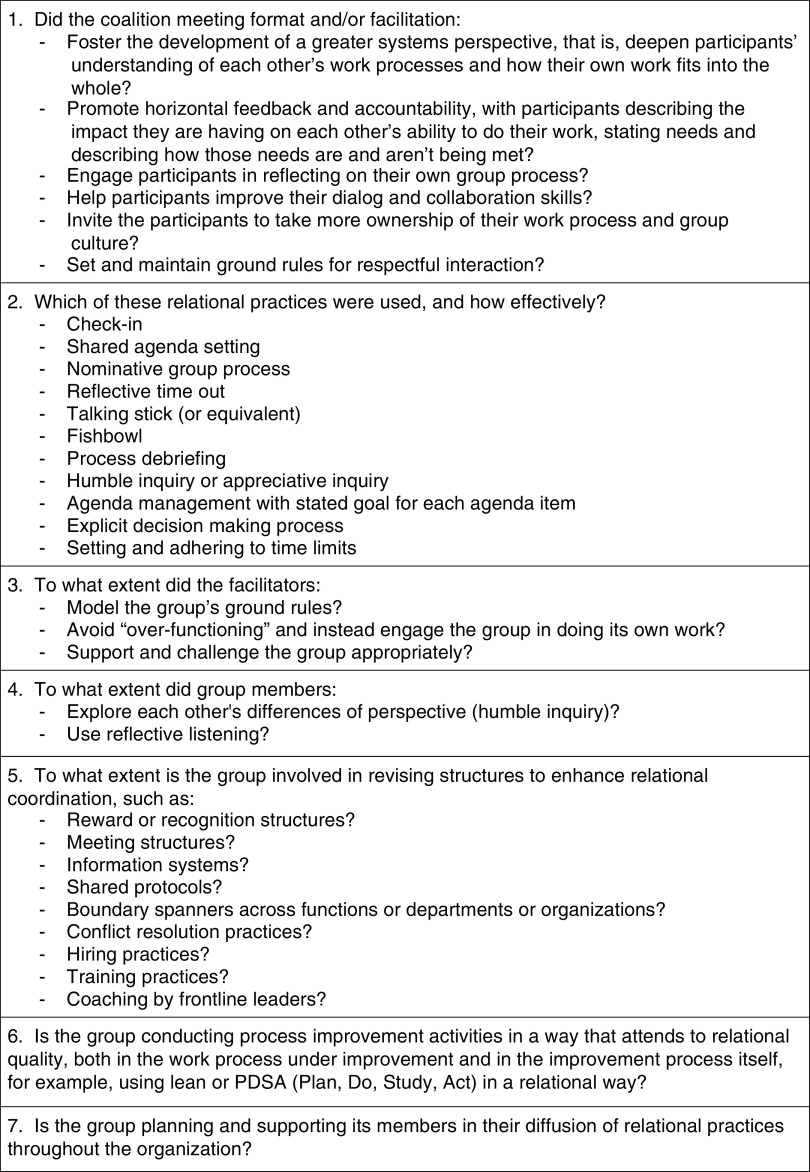
Relational coordination Intervention Fidelity Criteria.

The external consultant created a formal description of the intended cultural goals and the methods for achieving them to be used by the evaluators for assessing the fidelity of the actual intervention to the model (Fig. 3). Tufts CTSI leaders adapted the Fidelity Criteria into a simpler checklist they could use to prepare for each meeting.

## Data and Methods

### Formative Evaluation

We conducted a mixed-methods evaluation study to document implementation of the RC change process described above and to assess the impact of the intervention on the proximal outcome of work processes [17]. Because the Education, Research Services, and Clinical Trials Coalitions were the focus of the most intensive interventions, they received more focus in the evaluation.

### Sample

To assess changes in RC culture overall, a survey about RC considered personnel involved in the Education and Research Services work processes from both Tufts CTSI and from institutional offices which are not part of the CTSI. The sample size ranged from 74 to 94 participants, depending on wave of data collection. However, the additional research effort focused specifically on the 9 members of the Education, the 12 members of the Research Services, and 14 members of the Clinical Trials Coalitions. Study participants were heterogeneous with regard to their professional backgrounds and level of training and included faculty and staff involved in the delivery of all Tufts CTSI resources and services.

### Data Collection

Consistent with previous studies, the intervention was assessed using 4 types of data: logs by the intervention leaders about meeting practices, observations of meeting practices, individual interviews regarding progress of the intervention, and surveys of participants [18]. As is often the case with formative evaluations, early results, including findings from a survey about RC, were reported back to participants [19]. Sharing RC results with participants serves as a relational intervention that helps participants to identify opportunities that are typically invisible in most organizational contexts.

### Meeting Logs and Observations

During the first year, the coalitions met approximately monthly and then quarterly in subsequent years. The evaluation team sent trained participant observers to 1 meeting per coalition per quarter for a total of 10 observations in year 1 and 6 observations in year 2. Observers used a checklist to help capture facilitator and participant behaviors that reflected RC principles, taking notes by hand and gathering artifacts from the meetings such as agendas, work plans, and handouts.

### Individual Interviews

The evaluation team conducted 2 waves of individual interviews at the end of years 1 and 2. In the initial wave, the evaluation team selected 1 highly engaged and 1 less engaged participant from each coalition for a 30-minute phone interview. The semi-structured protocol asked respondents about meeting practices and the perceived value and outcomes of the process. It also invited their reflection on the interpretation of the RC principles (e.g., what does shared knowledge mean to you?). The second wave of interviews sought participants’ perceptions of changes in relational behaviors (e.g., knowledge of other people’s work), along with questions on whether or not participants use principles of RC in their Tufts CTSI work. Interviewers took notes during the call, but no tape recordings or direct quotations were used in order to reassure participants of confidentiality.

### RC Survey

The RC survey is a network measure in which respondents rate each workgroup that participates in a work process on 7 dimensions of RC using a 5-point Likert scale. The tool has been used previously in healthcare with good reliability (Cronbach α ranged between 0.85 and 0.95) and validity. It is particularly well-suited for measuring teamwork across multiple organizational boundaries [20].

Given the distributed nature of the CTSI’s work, a “not applicable” (NA) response option was added to the survey to allow respondents to indicate that they had no need to interact with a given work group. The Education RC survey included 7 workgroups and the Research Services RC survey had 16 workgroups present in all 3 waves of data collection. Cronbach α scores for RC ranged between 0.79 and 0.91.

### Team Climate Inventory

The Team Climate Inventory (TCI) [21] was adapted to measure groups’ climate for innovation, as well as to capture different aspects of team functioning. The TCI has been previously used in healthcare settings with good reliability (Cronbach α ranged between 0.84 and 0.94) and validity. Wording was modified to reflect the CTSI setting and a section was added to assess satisfaction with the coalition process. The resulting 52-item tool assessed coalition members’ views on participation, attitudes toward change and new approaches, views on the group’s objectives, internal monitoring and appraisal of the group’s work, and satisfaction with the organization of the group. In this sample, Cronbach α ranged between 0.91 and 0.96. A baseline TCI survey was administered shortly after the Coalition meetings were launched and a second TCI survey was administered 16 months later at the end of the official intervention.

### Data Analysis

Interview data were analyzed for key themes and written up as feedback memos. These memos were shared with intervention leaders to facilitate mid-course corrections and to validate key observations as well as support evaluation. RC and TCI survey data were summarized using basic descriptive statistics. For the RC survey, we considered both coalition members and respondents from the broader Tufts CTSI. Respondents with more than half the items marked NA were dropped from the analysis. To detect differences over the 3 waves we used 1-way analyses of variance (ANOVAs). The TCI survey included coalition members; analyses of differences between baseline and follow-up were limited to individuals who completed surveys for both waves using within-work group *t*-tests.

## Results

### Implementation Fidelity to RC Principles

As shown in Table 1, the intervention was initiated with a retreat on April 17, 2014. This was followed by the baseline RC survey and then two 3-hour retreats to review the survey results, explore interventions to improve RC and begin the development of action plans. The plan to create coalitions was developed by CTSI leaders based on the conversations at the retreats, particularly the recognized need for workgroups to have a better understanding of each other’s work (shared knowledge) and to improve the timeliness of communication. Each coalition was designed to be cross-functional and multidisciplinary. The coalitions were charged with working together to select and complete projects that improve their work environment and better serve the needs of CTSI “customers.”

**Table 1:** Timeline of key activities, 2014–2016

Key event	Date
Kick-off meeting	April, 2014
Relational mapping exercise	April, 2014
Relational coordination survey, W1	May, 2014
Two half-day retreats to review survey results	June, 2014
Coalition meetings begin meeting	September, 2014
Team climate inventory, W1	November, 2014
Relational coordination survey, W2	May, 2015
Year 1 individual interviews	June, 2015
Team climate inventory follow-up, W2	March, 2016
Relational coordination survey, W3	May, 2016
Year 2 individual follow-up interviews	November, 2016

W1, wave 1; W2, wave 2; W3, wave 3.

The newly formed coalitions met over the course of the study period. A number of new relational meeting practices were deployed and adherence to these practices was monitored using the Intervention Fidelity Criteria. The evaluation team found that relational meeting practices were carried out fairly consistently, with weaknesses in two areas: (1) the use of meetings as a vehicle for fostering a culture of collaboration and engagement, and (2) maintaining a culture of accountability.

Throughout the intervention a number of additional steps were taken to improve RC and support a more productive work environment. For example, Tufts CTSI established a repository of electronic “living documents” (e.g., policies, phone lists, organizational charts, etc.) in a shared online environment to improve access. Staff also implemented a new, internal, weekly newsletter that informed CTSI faculty and staff of personnel changes, reminders, events, and successes in a section called “Kudos and Congrats,” consistent with the structure *shared rewards*. An orientation for new staff was established and included RC and its 7 dimensions, consistent with the structural intervention *hiring and training for teamwork*. New staff were also invited to attend and participate in the Community Taskforce, a staff group that adopts the same meeting practices and RC principles as the coalitions. In addition, on the basis of the RC survey results, some workgroups received additional coaching to address specific within and between workgroup dynamics.

### RC

The wave 1 RC survey scores were similar for both work processes with a moderate overall RC score (Tables 2 and 3). The strongest dimensions were frequent communication and problem-solving versus blaming communication. The weakest RC dimensions for both work processes were timely communication and shared knowledge, similar to findings in other healthcare contexts.

**Table 2:** Relational coordination (RC) survey results for education survey by wave*

	Education Survey						
	Full sample				Education Coalition		
	Wave 1 mean score	Wave 2 mean score	Wave 3 mean score	*p*-Value†	Wave 1 mean score	Wave 2 mean score	Wave 3 mean score
Number	39	40	19		8	5	4
Response rate (%)	47	46	40				
1. Frequent communication	4.34	4.65	4.65	0.04	4.40	4.88	5.00
2. Timely communication	3.5	3.88	4.17	0.02	3.67	4.17	4.54
3. Accurate communication	3.75	4.3	4.35	0.005	4.05	4.60	4.87
4. Problem-solving communication	3.92	4.17	4.31	0.16	3.81	4.83	4.74
5. Shared goals	3.78	3.8	4.1	0.36	3.98	4.53	4.67
6. Shared knowledge	3.41	3.41	3.63	0.63	3.54	4.01	4.31
7. Mutual respect	3.72	4.04	4.21	0.05	3.71	4.71	4.71
8. Overall RC index	3.78	4.02	4.27	0.02	3.88	4.53	4.69

* Results shown in the table reflect scores for all Clinical and Translational Science Institute faculty and staff and institutional personnel who participated in the survey as well as collation members. The full survey includes individuals who were not members of the 3 coalitions. As a result, these scores reflect the broadest measure of culture change within the organization.

† To adjust for multiple comparisons, the Bonforroni correction for 8 tests makes the critical α=0.006 rather than 0.05.

**Table 3:** Relational coordination (RC) survey results for research services work group and the clinical trials and research services coalition by wave*

	Research Services Survey									
	Full sample				Clinical Trials Coalition			Research Services Coalition		
	Wave 1 mean score	Wave 2 mean score	Wave 3 mean score	*p*-value†	Wave 1 mean score	Wave 2 mean score	Wave 3 mean score	Wave 1 mean score	Wave 2 mean score	Wave 3 mean score
Number	55	54	55		7	4	6	5	6	5
Response rate (%)	47	48	64							
1. Frequent communication	4.34	4.28	4.3	0.93	4.4	4.57	4.51	3.99	4.33	4.77
2. Timely communication	3.62	3.72	3.72	0.78	4.13	3.58	3.77	3.51	3.49	3.77
3. Accurate communication	4.08	4.15	3.95	0.17	4.23	3.67	4.06	4.13	4.22	3.66
4. Problem-solving communication	4.1	4.13	4.1	0.87	4.14	4.06	3.95	3.69	3.88	4.31
5. Shared goals	3.96	4.12	4.02	0.56	3.83	3.88	3.78	3.81	3.69	4.06
6. Shared knowledge	3.29	3.47	3.41	0.74	4.5	3.31	3.4	3.19	3.43	3.85
7. Mutual respect	3.82	4.01	4.04	0.64	3.8	3.7	3.54	3.6	3.84	3.97
8. Overall RC index	3.84	3.89	3.89	0.91	4.01	3.81	3.85	3.62	3.78	4.05

* Results shown in the table reflect scores for all Clinical and Translational Science Institute faculty and staff and institutional personnel who participated in the survey as well as collation members. The full survey includes individuals who were not members of the 3 coalitions. As a result, these scores reflect the broadest measure of culture change within the organization.

† To adjust for multiple comparisons, the Bonforroni correction for 8 tests makes the critical α=0.006 rather than 0.05.

Changes in RC from baseline to the 2 follow-up assessments capture each respondent’s perspective of the entire work environment, not just of the specific coalition. For the overall Education work process, the largest significant change between waves was in accurate communication (Table 2). Timely communication, mutual respect, and overall RC also had positive gains; however, after adjusting for multiple comparisons, these were no longer significant (Table 2). For Education Coalition members, the RC scores tend to be higher than the organization-wide group and the size of change from wave 1 to wave 3 tends to be larger.

For the Research Services work process, the overall RC results showed few differences between waves. Mutual respect showed the greatest growth from 3.82 at wave 1 to 4.04 at wave 3 (Table 3), but was not statistically significant. Coalition members as a whole appear to have few differences over time. However, when distinguishing between the 2 coalitions that were part of the Research Services work process, the Research Services Coalition showed positive growth in frequent, timely, problem-solving communication, shared goals, shared knowledge, mutual respect, and overall RC between waves 1 and 3. In contrast, the Clinical Trials Coalition experienced flat or negative growth over time. Given the small sizes of these groups it was not possible to test for statistical significance.

### Team Climate Inventory

Eighteen staff completed both waves of the TCI. As shown in Table 4, on average, coalition participants were most positive about the group’s objectives. Views on participation and attitudes toward change were lower. All of these dimensions increased between waves 1 and 2, and changes in *views on objectives* and *satisfaction with the organization of the coalition* reached statistical significance despite a relatively small sample size.

**Table 4:** Team climate inventory results for individuals who completed 2 waves of data collection

	Number	Wave 1 mean score	Wave 2 mean score	W2−W1 difference	Within sample *p*-value*	Overall standard deviation
1. Participation in the coalition	19	3.34	3.56	0.22	0.14	0.67
2. Attitudes and change	18	3.40	3.74	0.34	0.03	0.69
3. Understanding objectives	18	4.86	5.34	0.48	0.002	0.78
4. Coalition monitors/appraises its work	17	4.04	4.62	0.58	0.02	1.49
5. Satisfaction with organization coalition	17	4.18	4.44	0.26	0.01	0.70

* To adjust for multiple comparisons, the Bonforroni correction for 5 tests makes the critical α=0.01 rather than 0.05. This means only views on group objectives and satisfaction with the organization of the coalition showed significant improvement.

### Interview Findings

The wave 1 interviews and observations revealed a high degree of mutual respect among coalition members and within the larger Tufts CTSI environment. Even when coalition members did not agree on a problem or solution, they did feel respected and able to express different viewpoints regardless of their role or training. This extended to “high-ranking doctors” who were perceived as willing to listen to other people’s ideas.

A much greater challenge was establishing the right meeting structure to foster collaboration and shared knowledge without requiring too much time. Participation in meetings was a struggle and individual attendance rates varied widely. Some people felt the coalition meetings were useful only if everyone participated, while others felt the coalition meetings had too much overlap and duplication with other meetings. As a result it was a challenge to have the right people at the table and to target participation to the task at hand.

Most participants were able to clearly identify key relational meeting practices including ground rules, “check-ins,” time-keeper roles, and appreciative debriefings. When asked if these practices were applied in their daily work beyond the coalition meetings, some said yes, it is common sense; others said that the spirit of the rules crept into their daily work in a positive way, but could not point to specific activities or changes they had made after participating in the coalition.

Each coalition was charged with selecting and completing a joint project during the intervention; for example a needs assessment/survey to support the development of a new certificate program (Education Coalition), a needs assessment for research training (Clinical Trials Coalition), and creation of a research services database and brochure (Research Services Coalition). All groups identified and completed 1 or more project and found them useful for their core CTSI work.

## Discussion and Implications

There is widespread agreement that more effective translation of biomedical research into health outcomes will require cultural, structural, and operational barriers to collaboration to be addressed. This challenge is generic to the entire biomedical research enterprise, and a particular focus of the NIH CTSA program. This paper describes the theory and implementation of an RC intervention to enhance translational research at Tufts CTSI.

The intervention reported here was of relatively low intensity. Two CTSI staff received 5–6 hours of coaching from an external consultant to prepare them to use coalition meetings to promote culture change and foster systems thinking, shared decision-making, responsiveness to other’s needs, lateral accountability, and process awareness. Although this external consultant supported the demonstration throughout implementation, much of the design and implementation was done by Tufts staff. Participation in the intervention (including cross-functional coalitions and task forces) was voluntary.

This formative evaluation found that those involved in the Education work process experienced increases in all dimensions of RC over the 2-year intervention, whereas participants in the Research Services work process experienced no overarching change in RC over this time period, although there appear to be coalition specific differences. The TCI, which focuses specifically on dynamics and innovation within the coalition teams themselves, showed a generally positive trend, particularly around satisfaction with group organization and views on objectives.

In addition to the modest changes in RC, the coalitions and taskforce teams successfully completed a number of joint work projects that were identified as valuable by their members. The resulting work products were facilitated by, or could not have been achieved without, cross-functional collaboration. Some work products were fairly simple to implement, but had not been previously completed due to unclear task ownership, lack of clear processes for maintenance, or time constraints. Other joint work products were complex and took months to implement.

Findings from this formative evaluation revealed some considerations for carrying out RC interventions in a CTSA. One major challenge was the commitments faculty leaders had outside of the CTSI (e.g., patient care, funded research, teaching, or administrative positions) making it difficult for them to invest in cross-functional relationships and to maintain the level of participation needed to sustain high shared knowledge and timely communication. Another concern was the burden that the additional meetings placed on already busy faculty and staff. Questions regarding the number of meetings emerged repeatedly in interviews. Even so, skeptical participants did not want to eliminate the meetings; rather they wanted to find a balance between too many and too few meetings. In the second year of the intervention, coalition meetings were reduced from monthly to quarterly which seemed to strike a better balance.

Two limitations of the evaluation itself deserve comment. First, the analysis of survey data was limited by the relatively small number of faculty and staff participating; this was particularly true for analysis of the TCI and RC survey within the coalitions. Second, CTSI performance outcome measures were newly implemented during the intervention period, making it difficult to assess the impact of the intervention on these measures.

There are a number of potential reasons why a stronger change in RC was not observed. Based on resource constraints, this project employed a strategy that vested responsibility for culture change in CTSI leaders who also had other substantial operational responsibilities and pressures. The gap between the observations of meeting practices and the Intervention Fidelity Criteria suggests the need for more intensive coaching of these leaders. Also, while a range of people participated in the coalitions, it was the staff, as opposed to faculty, who were often responsible for following through on action items. This disparity and the significant faculty commitments outside of Tufts CTSI may have been barriers to building a strong RC culture. Another challenge was the significant growth in the CTSI staff and staff turnover during the time period of the intervention. Finally, there was also a shift in mission for the CTSA program set by its funder (NIH) early in the intervention. As a consequence, defining shared goals and building shared knowledge became far more challenging, particularly for the faculty and staff tasked with implementing clinical trials, during the intervention than it was at the time of the baseline RC survey.

The difference in the RC results between the Education and Research Services work processes also suggests that the timeline required to achieve RC culture change may be longer when the work process is more complex. The Education work process had fewer and less diverse workgroups as well as a narrower scope than the Research Services work process, and over the 2-year intervention showed greater RC culture change with a continued upward trend in all RC dimensions. The Research Service Coalition appeared to be gaining the same type of momentum as the Education Coalition, but the Clinical Trials Coalition was faced with particularly challenging work objectives which were largely defined by the funding agency and required institutional infrastructure investments that were well beyond the scope of the coalition. These challenges made it harder for the Clinical Trials Coalition to identify appropriate and achievable goals and contributed to a sense of futility in coalition’s work.

In conclusion, a core task of CTSAs is to increase coordination and alignment among traditionally independent individuals and workgroups. We undertook a feasibility study of a multifaceted intervention based on RC theory. The intervention included relational and structural elements and appears to have produced some rather modest, but positive results, suggesting that culture change in clinical and translational science is indeed feasible and suggesting that there is more to be learned about the optimal design of such interventions.

## Study Highlights

One of the core challenges of a translational research enterprise such as an NIH CTSA organization is to coordinate the work of individual teams that often work independently, but serve the same research process. This study attempted to test whether an intervention based on the principles of RC can improve cross-functional collaboration. In addition to documenting the implementation process, the findings suggest modest success. Future work in this area should consider more targeted and sustained intervention models, in addition to drawing upon basic research on coordination mechanisms in complex systems.

## Authors Contribution

J.P. is responsible for study design, data analysis, and manuscript writing; A.R. is responsible for **s**tudy design, implementation, and data interpretation; L.W. is responsible for **s**tudy design and data interpretation; D.D. is responsible for **s**tudy design, implementation, and data interpretation; A.S. is responsible for study design, implementation, and manuscript writing; J.H.G. is responsible for study design, intervention, and manuscript writing; H.S. is responsible for implementation and data interpretation; J.B. is responsible for implementation, data collection, and analysis; S.M. is responsible for study design and implementation; H.S. is responsible for study design, implementation, manuscript writing, and funding.

## Declaration of Interest

The authors report that they have no conflicts of interest.
